# In Silico Analysis of the Age-Dependent Evolution of the Transcriptome of Mouse Skin Stem Cells

**DOI:** 10.3390/cells9010165

**Published:** 2020-01-09

**Authors:** L. Francisco Lorenzo-Martín, Xosé R. Bustelo

**Affiliations:** 1 Centro de Investigación del Cáncer, CSIC—University of Salamanca, 37007 Salamanca, Spain; Fran_lm@usal.es; 2 Instituto de Biología Molecular y Celular del Cáncer, CSIC—University of Salamanca, 37007 Salamanca, Spain; 3 Centro de Investigación Biomédica en Red de Cáncer (CIBERONC), CSIC—University of Salamanca, 37007 Salamanca, Spain

**Keywords:** skin homeostasis, epidermal stem cell, hair follicle, bulge, bioinformatics, time-course, transcriptomic analysis

## Abstract

The stem cells located in the hair follicle bulge area are critical for skin regeneration and repair. To date, little is known about the evolution of the transcriptome of these cells across time. Here, we have combined genome-wide expression analyses and a variety of in silico tools to determine the age-dependent evolution of the transcriptome of those cells. Our results reveal that the transcriptome of skin stem cells fluctuates extensively along the lifespan of mice. The use of both unbiased and pathway-centered in silico approaches has also enabled the identification of biological programs specifically regulated at those specific time-points. It has also unveiled hubs of highly transcriptionally interconnected genes and transcriptional factors potentially located at the core of those age-specific changes.

## 1. Introduction

The skin stem cells (SSCs) located in the bulge area of the hair follicles are important for replacing, restoring, and regenerating epidermal cells that have been lost, damaged, or become pathologically dysfunctional in postnatal periods [1,2]. These functions require the orchestration of both symmetrical and asymmetrical cell divisions to maintain the stem cell pool and generate the downstream cell lineage-committed precursors, respectively [3]. It was believed for a long time that SSCs were highly resistant to aging [4]. Consistent with this, the total numbers of SSCs do not significantly change between adult and aging mice [5]. However, more recent analyses have revealed that the SSCs from aging mice become dysfunctional, showing significant reductions in stemness properties when tested in either cell culture or in vivo [6]. This has been connected to SSC extrinsic factors, such as inflammation-derived signals from the surrounding niche [6].

The SSCs have been extensively characterized at the transcriptomal level both in toto and in single cell-based experiments in mice [7,8]. This has led to a good understanding of the gene expression programs linked to cell stemness, the quiescent and active state of SSCs, cell lineage differentiation events, and oncogenically transformed states [8,9,10,11,12]. Likewise, recent studies have also unveiled the transcriptional programs associated with the aging of SSCs [6]. All these studies have been concentrated in very specific age periods of mice. However, to our knowledge, there are no available data regarding the evolution of the SSC transcriptome during consecutive age periods. As a result, we do not know whether that transcriptome fluctuates in an age-dependent manner and, if that were the case, the specific age intervals in which such changes take place. Likewise, we do not have a clear idea of the precise timing in which the molecular features of aging SSCs begin to emerge in mice. To address this issue, we decided to carry out microarray analyses in flow-cytometry purified SSCs from six different age periods of mice. These groups ranged from young (18-day-old) to old (12-month-old) ages. We combined these analyses with the use of multiple bioinformatics tools to unveil any changes in gene expression and biological programs taking place across those experimental time-points. Using this approach, we found that the SSC transcriptome is highly dynamic throughout the age periods interrogated. We also unveiled biological functions, coregulated pathways, and transcriptional hubs that are associated with those age-dependent gene expression changes. Finally, our data also suggest that the molecular features of aging SSCs are already detected at much earlier times than previously anticipated.

## 2. Methods

### 2.1. Animal Studies

Male C57BL/6J mice were maintained and utilized according to protocols approved by the Bioethics Committee of the University of Salamanca. Mice were kept in ventilated rooms in pathogen-free facilities under controlled temperature (23 °C), humidity (50%), and illumination (12-h-light/12-h-dark cycle) conditions.

### 2.2. Isolation of CD34^+^ Itgα6^+^ SSCs

This procedure was carried out as previously described [13]. Briefly, the back skin of animals was excised, cleaned, and digested in 0.25% trypsin (ThermoFisher, Waltham, MA, USA; Catalog No. 25200056) overnight. The cell suspension was then filtered, resuspended in EMEM (Lonza, Basel, Switzerland; Catalog No. BE06-174G) supplemented with 15% fetal bovine serum (ThermoFisher, Catalog No. 10500064), incubated for 30 min on ice with biotin-conjugated antibodies to CD34 (dilution 1:50, eBioscience, San Diego, CA, USA; Catalog No. 13-0341-85) and, subsequently with both APC-conjugated streptavidin (dilution 1:300, BD Biosciences, Franklin Lakes, NJ, USA; Catalog No. 554067) and PE-conjugated antibodies to CD49f (dilution 1:200, AbD Serotec, Kidlington, UK; Catalog No. MCA699PE) for 30 min. The cell suspension was then incubated with 4′,6-diamidino-2-phenylindole (DAPI) (0.1 ng/µL, Sigma-Aldrich, Saint Louis, MO, USA; Catalog No. D9542) for 5 min to mark and exclude dead cells. Finally, positive cells for both CD34 and CD49f were isolated using a FACSAria III flow cytometer (BD Biosciences) and analyzed with the FlowJo software (Ashland, OR, USA).

### 2.3. RNA Extraction and Transcriptome Profiling

Flow cytometry purified CD34^+^ Itgα6^+^ SSCs were lysed in RLT buffer (QIAGEN, Hilden, Germany; Catalog No. 74004) and total RNA was extracted using the QIAGEN RNeasy Micro Kit (QIAGEN, Catalog No. 74004) according to the manufacturer’s instructions. Purified RNA was processed exactly as indicated elsewhere [14] and analyzed using GeneChip Mouse Gene 1.0 ST microarrays (Affymetrix, Santa Clara, CA, USA). R and Perl languages were used to perform the bioinformatic analyses. Signal intensity values were obtained from CEL files after applying the Robust Multichip Average (RMA) function from the ‘affy’ package for background adjustment, quantile normalization, and summarization [15]. For the visualization of expression and enrichment data, a 0–1 normalization was used:

(1) $$x=\frac{x-x_{min}}{x_{max}-x_{min}}$$

### 2.4. Establishment of Gene Expression Patterns

We used both Chi-squared and fold change thresholds to distinguish probesets with dynamic and stable behavior along the time-points interrogated as reported before [16]. Briefly, for each probeset, a Chi-squared test with N–1 degrees of freedom was applied as follows:

(2) $$\begin{array}{ccc} \chi^{2}=\overset{n}{\underset{i}{\sum }}\frac{{\left(\bar{X_{i}}-\;\bar{X}\right)}^{2}}{\bar{X}} & =\frac{{\left(\bar{X_{0.6}}-\;\bar{X}\right)}^{2}}{\bar{X}}+\frac{{\left(\bar{X_{1}}-\;\bar{X}\right)}^{2}}{\bar{X}}+\frac{{\left(\bar{X_{2.5}}-\;\bar{X}\right)}^{2}}{\bar{X}}+\frac{{\left(\bar{X_{4}}-\bar{X}\right)}^{2}}{\bar{X}}+\frac{{\left(\bar{X_{6}}-\bar{X}\right)}^{2}}{\bar{X}} & +\frac{{\left(\bar{X_{12}}-\;\bar{X}\right)}^{2}}{\bar{X}} \end{array}$$

where $\bar{X_{i}}$ is the mean expression of the gene for the triplicates for each time-point *i*, $\bar{X}$ is the overall mean expression across all time-points, and *n* is the number of time-points. For probesets with adjusted *P*($\chi^{2}$) < 0.01, a > 2-fold change requirement was empirically established. The identification of time-course expression profiles was done by soft clustering using the Mfuzz R package [17]. After standardization of the expression values for Euclidian space clustering, we applied the *mfuzz* function with a fuzzifier value of 1.25 and a ranging number of cluster centers to determine the optimal number of non-overlapping expression patterns. For the inclusion of a probeset in a particular cluster, the membership value threshold was set to 0.5.

### 2.5. Gene Set Annotation and Enrichment

Functional annotation of gene sets was performed using Metascape [18] and ToppFun [19] for biological processes and molecular functions, respectively. An FDR q-value of 0.05 was set as threshold for statistical significance. ssGSEAs were used to calculate the fitness of different hallmark, gene ontology, and epidermal stem cell signatures across time [20,21]. Hallmark and gene ontology signatures were obtained from the Molecular Signatures Database [22]. SSC-related gene signatures were obtained from previous studies [10,23]. The time-course enrichment scores for these signatures were used to build the signature correlation matrix, calculated through *corrplot* (https://cran.r-project.org/web/packages/corrplot). Correlations were considered statistically significant when the Pearson coefficient had *p* values below 0.05. Functional clusters were established when every pairwise correlation within a group of signatures was found significant. For the discovery of transcription factor binding motifs in the promoters of the coregulated genes, the iRegulon software was used [24]. A collection of 9713 position weight matrices (PWMs) was applied to analyze 10 kb centered around the TSS. With a maximum false discovery rate (FDR) on motif similarity below 0.001, we performed motif detection, track discovery, motif-to-factor mapping, and target detection.

### 2.6. Weighted Correlation Network Analyses

In order to identify the transcripts at the core of every gene expression pattern, the WGCNA R package was used [25]. To this end, we constructed each weighted gene network from the corresponding expression matrix through the *blockwiseModules* function. The *pickSoftThreshold* function was used to select the soft thresholding power according to network topology. Consensus module detection within each expression pattern was omitted and kept to one module as the number of clusters had been already optimized. The heatmap plot depicting the adjacency matrix was created with the *TOMplot* function. To calculate the intramodular connectivity for each gene, we computed the whole network connectivity for each expression pattern through the *intramodularConnectivity* function. Hubs were defined as the 10% most connected genes within each expression pattern. The known functional interactions among hubs were obtained through the String tool [26]. Cytoscape software was used to perform network data integration and visualization [27].

### 2.7. Data Availability

Microarray data reported in this paper was deposited in the GEO database (https://www.ncbi.nlm.nih.gov/geo/) under the accession number GSE137176.

## 3. Results

### 3.1. The SSC Transcriptome Fluctuates in an Age-Dependent Manner

**Figure 1:**
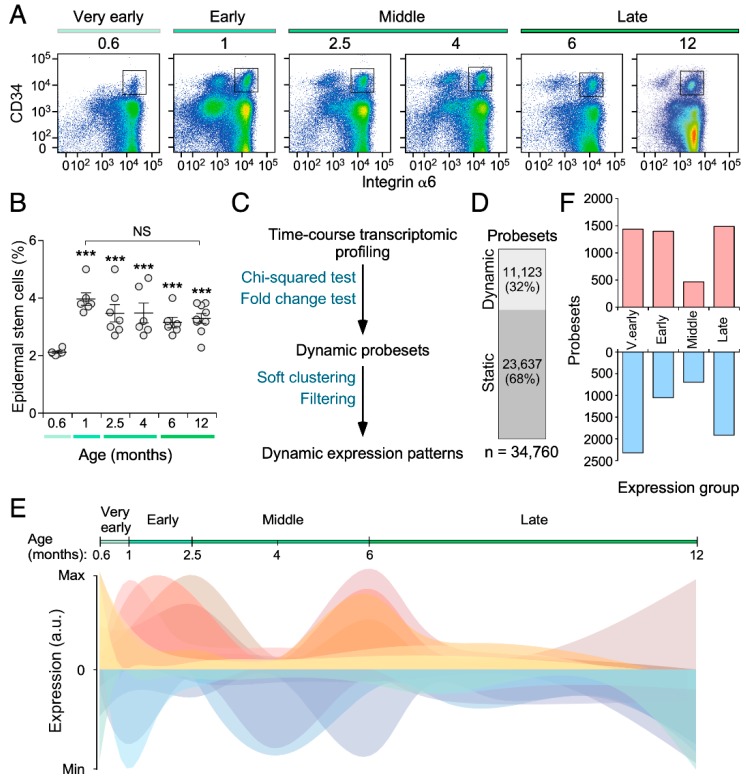
The transcriptome of skin stem cells fluctuates in an age-dependent manner. (**A**) Representative flow cytometry scatter plots showing the different epidermal populations according to CD34 and integrin α6 expression at the indicated age points (top). The classification in “very early” (18-day-old), “early” (30-day-old), “middle” (2.5- to 4-month-old), and “late” (6- to 12-month-old) populations is also indicated (top). The gate used for the isolation of basal bulge epidermal stem cell population is depicted with a square. (**B**) Quantitation of the abundance of the CD34^+^ Itgα6^+^ SSC pools in the skin of mice at the indicated ages. ***, *p* < 0.001; NS, not statistically significant (ANOVA and Dunnett’s tests, *n* = 6 (18-day-old mice), 6 (30-day-old mice), 7 (2.5-month-old mice), 6 (4-month-old mice), 6 (6-month-old mice), 9 (12-month-old mice). The correlation with the age intervals (“very early”, “early”, “middle”, “late”) is indicated with colored bars as in (A) (bottom). Data represent the mean ± SEM. (**C**) Scheme representing the bioinformatic pipeline followed to identify dynamic expression patterns of CD34^+^ Itgα6^+^ SSCs. (**D**) Distribution of the 34,760 probesets of the Affymetrix Mouse Gene 1.0 ST array between the dynamic and static groups. (**E**) Graphical representation of the time-dependent fluctuation of the 14 nonredundant expression patterns of SSCs described in the main text. (**F**) Number of dynamic probesets found in the “very early” (V. early), “early”, “middle”, and “late” age time-points. Bar color indicates positively (red) and negatively (blue) expression enrichment of probesets against the baseline.

To approach the age-dependent evolution of the transcriptome of mouse SSCs, we first purified by flow cytometry CD34^+^ Itgα6^+^ SSC pools from 0.6- (“very early” age time-point), 1- (“early” time-point), 2.5-, 4- (“middle” time-points), 6- and 12-month-old (“late” time-points) mice (Figure 1A). The first time-point selected corresponds to the earliest stage in which the CD34^+^ Itgα6^+^ SSCs can be clearly identified using flow cytometry approaches (Figure 1A). During the cell purification procedure, we observed that the bulge CD34^+^ Itgα6^+^ SSC population expands between the 18 to 30 postnatal days in the skin of mice (Figure 1A,B). However, as expected from previous reports [5], the numbers of those cells do not undergo any further statistically significant change thereafter (Figure 1A,B). Upon microarray analyses of each purified cell population, the expression profiles obtained were processed using a computational approach designed to identify transcripts with either dynamic or static expression patterns in the six interrogated age periods (for details, see Methods) (Figure 1C). These analyses revealed that 32% of the transcripts present in SSCs displays a dynamic behavior according to both Chi-squared distribution and change in expression (≥2-fold) criteria (Figure 1D).

**Figure 2:**
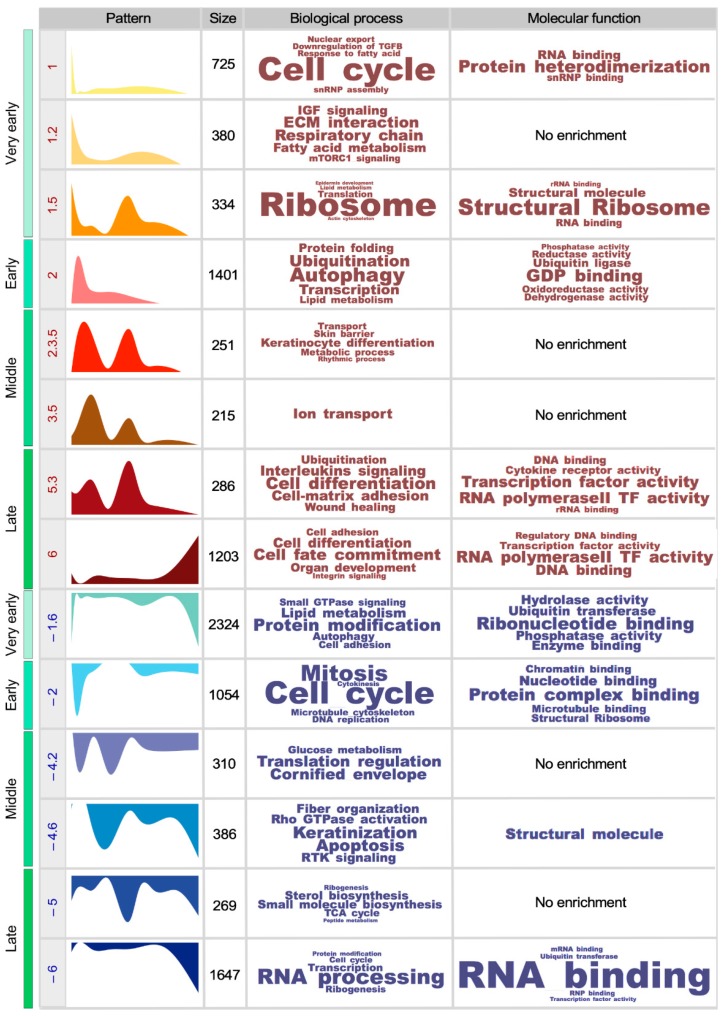
Time-driven expression patterns in mouse SSCs. Scheme showing the 14 nonredundant dynamic expression patterns found in this study. The table includes from left to right: (i) the experimental time-point (very early, early, middle, late) in which the indicated gene expression pattern is detected. (ii) The peak position (1 = 18-day-old, 2 = 30-day-old, 3 = 2.5-month-old, 4 = 4-month-old, 5 = 6-month-old, 6 = 12-month-old). (iii) The graphic representation of the indicated gene expression cluster. (iv) The number of probesets associated with the indicated gene clusters. (v) The top-enriched biological processes (with term size proportional to the −Log(*P*) obtained for each of them). (vi) The top-enriched molecular functions (with term size proportional to the −Log(*P*) obtained for each of them).

Further soft-clustering analyses revealed that these dynamic genes can be subdivided into 14 nonredundant gene expression subsets according to their respective age-dependent patterns of expression (Figure 1E and Table S1, see also Figure 2 further below). These subsets show either single or bimodal patterns of expression depending on whether they are detected specifically in a given time-point or in several age intervals, respectively (Figure 1E and Figure 2 below). The bimodal ones also differ among themselves depending on the total number of differentially expressed genes detected at each time-point (Figure 1E and Figure 2 below). We also found that the larger transcriptomal changes are detected at the “very early”, “early”, and “late” age periods (Figure 1F). By contrast, they are less relevant in the case of the “middle” age intervals (Figure 1F). There is also a larger percentage of downregulated than of upregulated probesets in most age time-points but the “early” one (Figure 1F). Collectively, these results show that the transcriptome of the CD34^+^ Itgα6^+^ SSC pool undergoes significant fluctuations in an age-dependent manner.

### 3.2. The Functionality of the SSC Transcriptome Drifts over Time

We next utilized the Metascape [18] and ToppFun [19] bioinformatics tools to identify in an unbiased manner the biological and molecular processes associated with the 14 gene expression subsets described in Figure 1. We found that two of the upregulated gene expression subsets (#1 and #1.2) found in the “very-early” time-point encode proteins linked to the cell cycle (#1), the respiratory chain (#1.2), extracellular matrix interactions (#1.2), IGF (insulin growth factor) signaling (#1.2), and fatty acid metabolism (#1.2). The third upregulated gene subset (#1.5) seen in this age period includes functions connected to ribosome biogenesis and translation. This bimodal subset is also detected in the SSCs from the “late” age time-point interrogated in our study (Figure 2 and Figure S1, Table S1). This spectrum of functions is probably associated with the expansion in the numbers of SSCs observed between postanal days 18 and 30 (Figure 1B). In the case of downregulated genes, we only found a single gene expression subset (#−1.6) that fulfills our statistical criteria. This subset is composed of transcripts linked to protein modification, lipid metabolism, autophagy, cell adhesion, and small GTPase signaling (Figure 2 and Figure S1, Table S1). This gene expression subset becomes downmodulated again in the latest experimental time-point (SSCs from 1-year-old mice) (Figure 2 and Figure S1, Table S1).

The “early” transcriptional time-point is much less complex, since it is only composed of two subsets of differentially expressed genes (#2, #−2) (Figure 2 and Figure S1, Table S1). Subset #2 harbors upregulated transcripts linked to autophagy, ubiquitinylation, transcription, protein folding, and lipid metabolism (Figure 2 and Figure S1, Table S1). Subset #−2 includes downregulated mRNAs associated with proliferation-related processes (Figure 2 and Figure S1, Table S1). This is in line with the observation that the expansion of numbers of SSCs is basically concentrated in the transition from the “very early” to the “early” time-points (Figure 1B).

The SSCs from the “middle” age time-points (2.5- to 4-month-old mice) are more stable from a transcriptomal point of view than those from the earlier time-points (Figure 1F and Figure 2, Table S1). The two upregulated gene expression signatures showing dynamic expression patterns at this stage (#2.3.5 and #3.5) are predominantly involved in keratinocyte differentiation-, metabolic-, skin barrier-, rhythmic- (all in subset #2.3.5), and ion transport-related (subset #3.5) processes (Figure 2 and Figure S1, Table S1). These transcripts become upregulated again in the SSCs from 6-month-old mice (Figure 2). The two downregulated gene expression subsets (#−4.2 and #−4.6) found in this period show enrichments in mRNAs associated with keratinization (#−4.6, #−4.2), apoptosis (#−4.6), Rho GTPase and receptor tyrosine kinase signaling (#−4.6), fiber organization (#−4.6), translation regulation (#−4.2), and glucose metabolism (#−4.2). The genes belonging to subset #−4.6 become downmodulated again in the SSCs from 1-year-old mice (Figure 2).

The transcriptional programs associated with the “late” age time-points regain in complexity both in terms of the number of differentially expressed gene subsets (two upregulated, two downregulated) and the total count of genes involved (Figure 1F and Figure 2). Their pattern of expression is also complex, since they include genes whose expression is specifically altered in SSCs from 6- (subsets #5.3 and #−5) and 12-month-old (subsets #6 and #−6) animals (Figure 1E and Figure 2, Table S1). The functions associated with the upregulated subsets include cell fate commitment, cell differentiation, organ development, cell adhesion, interleukin signaling, wound healing, and ubiquitinylation (Figure 2 and Figure S1, and Table S1). Many of these biological fingerprints have been described before in SSCs from aging mice (>18-month-old) [6], suggesting that the aging-like phenotype is acquired earlier than previously anticipated. The gene expression subsets that are downregulated during this period also suggest that the SSCs have reduced proliferation activity (Figure 2 and Figure S1, Table S1). Other molecular features associated with each of the foregoing gene expression subsets are summarized in Figure 2 and Figure S1, Table S1.

### 3.3. Key SSC-Related Signaling Pathways are also Age-Sensitive

**Figure 3:**
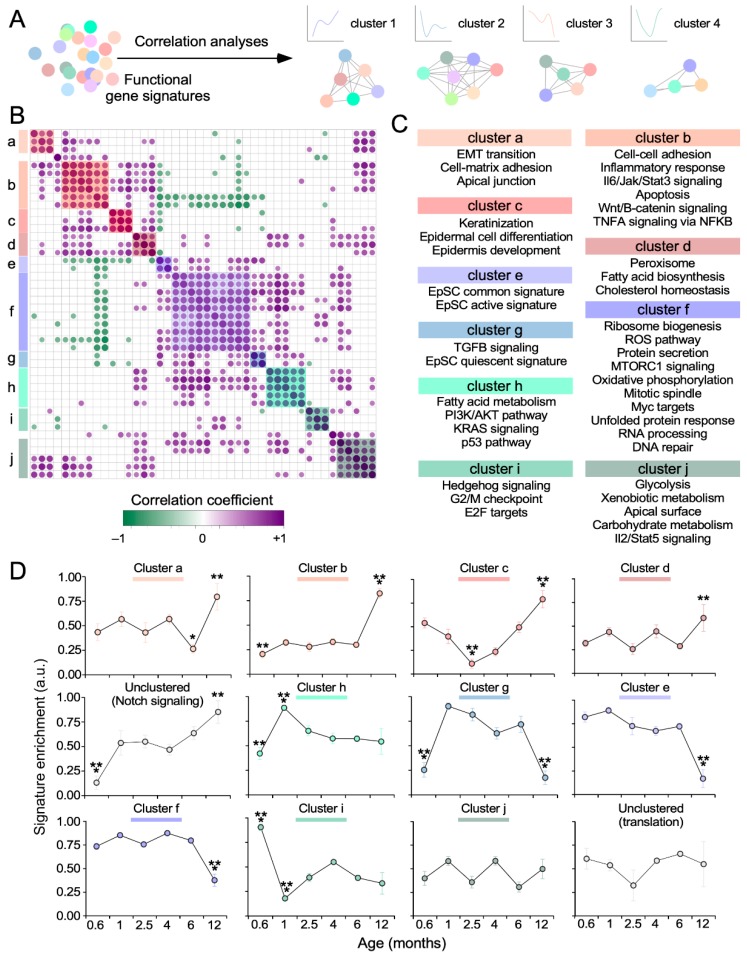
Key SSC-linked signaling pathways are age-sensitive. (**A**) Scheme representing how highly coexpressed pathways are assigned to specific coexpression clusters. Correlation matrix (**B**) and functional clusters (**C**) obtained with the in silico strategy indicated in (A). Only correlations with *p* values below the significance threshold of 0.05 were considered. Positive and negative correlations are shown in purple and green, respectively. Dot size and color reflects the magnitude of the Pearson correlation coefficient (*r*). Coexpression clusters are shaded in color (B,C). (**D**) Plots showing the enrichment pattern across time of the different pathway clusters identified in panel B. *, *p* < 0.05; **, *p* < 0.01; ***, *p* < 0.001 (Mann–Whitney test). Data represent the mean ± SEM.

To further characterize the biological programs associated with the age-specific gene expression subsets identified in this study, we decided to utilize single-sample gene set enrichment analyses (ssGSEA) to monitor the age-dependent evolution of hallmark pathways and gene signatures directly associated with SSC function [10,23]. The time-course enrichment scores obtained in these analyses were then used to build signature correlation matrices using the *corrplot* algorithm (see Methods) (Figure 3A). With this strategy, we expected to obtain a dynamic view of the age-dependent coevolution of pathways intrinsically associated with SSC functions that could be overshadowed when using the unbiased functional annotation analyses (Figure 3A).

These analyses revealed the presence of 10 clusters of pathways whose expression is coregulated (Figure 3B,C) in the SSCs obtained from specific age time-points (Figure 3D). Cluster “i”, which encompasses signatures for E2F targets, G_2_/M checkpoint elements, and Hedgehog signaling (Figure 3B,C), is the only one showing a specific enrichment in the “very early” age time-point (Figure 3D). This cluster becomes downregulated in the “early” time-point (Figure 3D), thus corroborating the previous changes in the expression of proliferation-related genes that were seen in those two periods using functionally unbiased in silico approaches (Figure 2). These results also agree with the known role of the Hedgehog pathway in driving the activation of SSCs [3]. Cluster “h”, which is composed of a variety of signaling (K-Ras, PI3K/Akt), Tp53, and fatty acid-related metabolic pathways (Figure 3B,C), becomes downmodulated and upregulated during the “very early” and “early” time-points of our study, respectively (Figure 3D). We could not detect any cluster specifically upregulated in the “middle” time-points (Figure 3D). However, cluster “c” (which is mostly composed of cell differentiation-linked programs) is specifically downregulated in that period (Figure 3D). These analyses also revealed that the majority of coregulated functional clusters becomes enriched at the “late” age time-points (e.g., clusters “a” to “d”, Figure 3D). The Notch pathway also belongs to this category (Figure 3D), although its signature does not show any statistically significant coregulated expression with any of other gene groups (data not shown). These coregulated pathways are associated with cytoskeletal and/or epithelial-mesenchymal transition responses (clusters “a” and “b”), inflammatory programs (cluster “b”), Wnt/β-catenin and TNFα signaling (cluster “b”), cell differentiation (cluster “c”), and metabolism (cluster “d”). These results match well the biological programs that were picked up when using the unbiased functional annotation methods (Figure 2), further reinforcing the idea that aging-associated SSC programs are activated well before the SSCs become dysfunctional in aging animals. Finally, we found clusters that become coordinately downregulated at the “very early” (clusters “b” and “h” and Notch, see above), “early” (cluster “i”, see above), “middle” (cluster “c”, see above), “very early” plus “late” (cluster “g”), “early late” (cluster “a”, see above), and “very late” (clusters “e” and “f”) time-points (Figure 3D). Cluster “e” contains SSC signatures associated with stemness and activation (Figure 3C). Cluster “f” encompasses signatures related to proliferation, signaling, and metabolic programs (Figure 3C). Cluster “g” harbors gene signatures connected to TGFβ signaling and SSC quiescence (Figure 3C). It is also worth noting that we found gene signatures that show coregulation without undergoing differential expression such as, for example, those included in cluster “j” (which contains signatures associated with a number of metabolic programs, apical surface polarity, and interleukin2-Stat5 signaling) (Figure 3C,D).

In terms of expression behavior, the coregulated functional clusters can be classified in three main categories (Figure 3D): (i) clusters displaying single and homogeneous changes in expression (upregulation or downregulation) in specific age time-points (e.g., clusters “d, “e”, “f”). (ii) Clusters with antagonistic behavior (repression and upregulation) depending on the time-point selected (e.g., clusters “a”, “b”, “c”, “h”, and “i”, Notch). (iii) Clusters with homogeneous changes in expression in different time-points (e.g., cluster “g”). Taken together, these results further support the idea that the transcriptional program of SSCs varies significantly along the life of mice. They also indicate that aging-like programs are switched on well before the SSC dysfunctions become apparent in these animals.

### 3.4. Identification of Age-Dependent Transcriptional Hubs in SSCs

**Figure 4:**
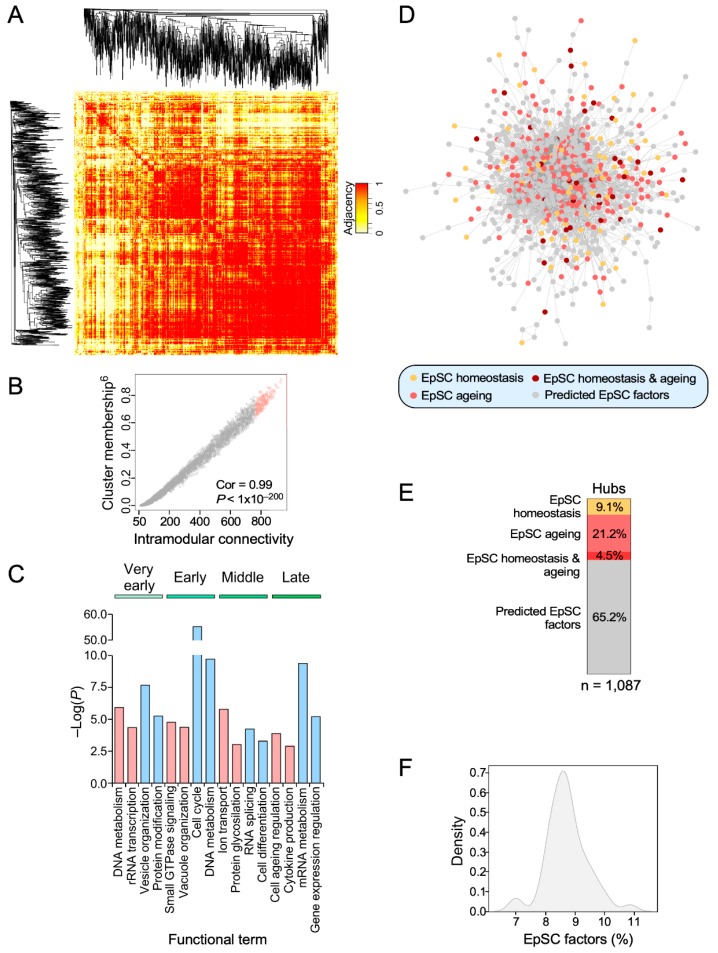
Identification of age-dependent mRNA-based hubs in SSCs. (**A**) Representative example of the adjacency heatmaps computed for each expression pattern using the WGCNA R package. Red areas indicate the presence of probesets that are highly correlated transcriptionally. Probeset clustering according to adjacency is depicted on the sides of the heatmap. (**B**) Representative example of a scatter plot showing the direct relationship between the cluster membership and the intramodular connectivity scores among the probesets within each expression pattern. The Pearson correlation coefficient with the corresponding *p* value is indicated. Identified hubs are highlighted in color. (**C**) Main functional terms derived from the gene ontology analysis of the identified hub genes for each expression pattern. Positive (“+”) and negative (“−”) enrichments against baseline are indicated. (**D**) Association network of the hubs identified in this study. Hubs that have not been previously associated with SSC homeostasis and/or ageing are indicated in gray color. (**E**) Percentage of the hubs identified in this study that have been associated with SSC homeostasis and/or ageing. Color coding is the same as in (**D**). (**F**) Density plot showing the percentage of genes associated to SSC homeostasis and/or ageing that are contained in random sets of 1087 genes (*n* = 50).

We next implemented a series of in silico approaches to identify genes that, given their high transcriptional interconnectivity with the rest of dynamic transcripts identified in our study, could be potentially involved in the age-dependent regulation of the SSC transcriptome. This idea stemmed from previous observations indicating that the most interconnected nodes (hubs) within specific signaling and transcriptomal networks usually play relevant roles in the orchestration of such biological programs [28,29]. To this end, we performed weighted correlation network analyses to calculate both the adjacency and the intramodular connectivity of all the genes that belong to the age-dependent gene expression subsets previously identified in this study (Figure 2 and Figure 4A, Table S2). Such parameters are highly related to the eigengene-based cluster membership score of each transcript (Figure 4B). To further increase the stringency of these analyses, we defined as hubs only the 10% of the top-connected nodes found in each age period-specific expression pattern (Figure 4B and Table S2). As a proof of principle of the validity of this approach, we found that the small hub gene collection derived from this analysis (1087 genes) shares functional overlap with the biological programs identified using the whole gene signature analyses (compare Figure 2 and Figure 4C). In particular, these hubs recapitulate the proliferative and non-proliferative status of SSCs that were found before during the “very early” and “early” time-points, respectively (Figure 4C). They also display the cell aging and inflammatory features previously seen in SSCs from the “late” age periods (Figure 4C). These hub-based analyses also picked up functions that had been overlooked by the previous functional annotation approaches such as, for example, the regulation of vesicle organization and RNA splicing at some age periods (Figure 4C). As a negative control, randomly picked genes accounting for 10% of each age time-point-specific gene signature cannot identify any of those functions (data not shown). To further validate the relevance in these hubs in epidermal stem cell context, we compared our data with those from previous studies that have described key factors associated with SSC homeostasis and ageing [6,9,10]. These analyses revealed that 34.8% (378 genes) of the genes present in our hub collection had been previously associated with SSC homeostasis (Figure 4D,E). As control, sets of 1087 randomly chosen genes show a much lower overlap (8.6% of genes) in the same analysis (Figure 4F).

### 3.5. Identification of Transcriptional Factors Potentially Involved in the SSC Transcriptome

**Figure 5:**
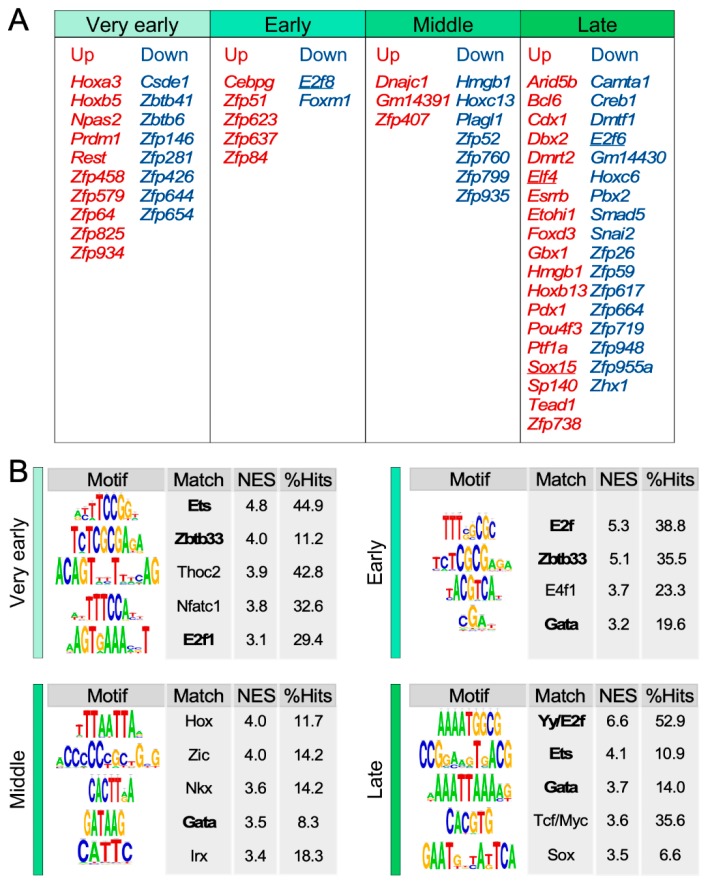
The dynamic expression patterns of SSCs are enriched in binding sites for specific transcription factors. (**A**) Up- (red) and downregulated (blue) transcriptional factors identified as hubs in the indicated experimental age intervals. Transcriptional factors also detected in the analyses shown in (**B**) are underlined. (**B**) Enriched transcription factor binding sites in the promoters of the hubs associated with each time-point gene expression program. The sequence logos, normalized enrichment score (NES), and the percentage of hits among the hubs are indicated. Transcriptional factors found in several age-points are depicted in bold.

Finally, we focused on the identification of transcriptional factors potentially involved in the age-dependent changes observed in the transcriptional programs of SSCs. To this end, we followed a two-pronged in silico approach: (i) the identification of the transcriptional factors that behave as hubs according to our previous bioinformatics analyses (Figure 4D and Table S2). (ii) The identification of transcriptional factors whose binding sites are enriched in the promoter regions of the SSC gene hubs using the iRegulon software [24] (see Methods). The former approach identified a number of mRNAs encoding transcriptional factors that are specifically regulated in each of the age time-points used in our study (Figure 5A). The larger subsets of this functional category are associated with the “very early” and “late” stages, the time intervals that exhibit the largest variations in the number of differentially expressed genes according to our prior analyses (Figure 1F).

This analysis also revealed a high percentage of transcriptional factors belonging to the zinc finger protein (Zfp) family that are present in most hub subsets and age periods (Figure 5A). The function of most of these proteins is largely unknown. Additionally, with the exception of some specific hubs (E2F, Ets, and Sox family members) (Figure 5A,B), we could not find any significant enrichment of the binding sites of all those transcriptional factors in the promoter regions of the rest of hubs identified in our analyses. However, it is worth noting that, due to their poor functional characterization, the information on 66% of all those factors is not contained in the datasets commonly used for the in silico identification of transcriptional factor DNA binding sites.

The second in silico approach did find the statistically significant enrichment of DNA binding sites for specific subsets of transcription factors in the promoter regions of the age-regulated gene hubs (Figure 5B). In the case of the signatures specifically expressed in the “very early” time-points, we found an enrichment in binding sites for members of the Ets family, Zbtb33, Thoc2, NFATc1, and E2F1 (Figure 5B). Many of these factors have been previously linked to either proliferation (Ets, E2F) or stem cell self-renewal [30,31,32,33]. Interestingly, the binding sites for some of those factors also become apparent in other experimental time-points. For example, the enrichment for Zbtb33 and E2F family DNA binding sites is also observed in the promoters of hubs that show changes in expression in the “early” age points (Figure 5B). This can be connected with the transcriptional repression seen at the “early” time-point according to all our previous in silico analyses (Figure 2, Figure 3C,D, and Figure 4C). The enrichment of Ets DNA binding sites is also seen in the case of the promoters of “late” period hubs (Figure 5B), an observation that could be also associated with the downmodulation of proliferation-related programs that was detected in this period with our independent in silico analyses (Figure 2 and Figure 3C,D).

In addition to E2F and Zbtb33, the promoter regions of the hubs specific for the “early” experimental phase harbor DNA binding sites the polycomb protein E4F1 and GATA family factors (Figure 5B). Remarkably, E4F1 plays key roles in skin stem cell maintenance probably in connection with the Bmi1-Arf-Tp53 pathway [34]. This finding is also consistent with the presence of Tp53 pathway-associated gene signatures in the cluster “h” of coregulated genes found in this period (Figure 3C,D). The role of GATA proteins in epidermal stem cells is less characterized, although one member of this family, GATA3, has been involved in cell lineage determination in the skin in association with Let1 and Wnt proteins [35]. Different types of DNA binding sites for GATA proteins are also observed in all the subsequent age periods analyzed in our work (Figure 5B). The hubs with changes of expression in the “middle” age period are enriched in binding sites for GATA and other poorly characterized factors in the context of stem cell biology such as Hox, Zic, Nkx, and Irx family proteins.

The promoter regions of the “late” period hubs are enriched in DNA binding motifs for E2F, ETS, GATA, the Wnt/β-catenin effectors (TCF/Myc), and Sox family proteins (Figure 5A,B). These data are consistent with the downregulation of proliferation-related genes (E2F, ETS) (Figure 2 and Figure 3C,D), the acquisition of more differentiated and aging features (GATA, Sox, Wnt) [35,36,37,38] (Figure 4C), and the upregulation of Wnt/β-catenin gene signatures observed in our previous in silico analyses (Figure 3C,D; cluster “b”). However, given that the Myc gene signatures are downmodulated in this period (Figure 3C,D; cluster “f”), it is likely that the Wnt target genes could be regulated by either TCF proteins or other transcriptional factors working downstream of Wnt.

## 4. Discussion

The characterization of the gene expression fluctuations along step-wise experimental time-points requires the combination of multiple in silico strategies in order to unwrap the variety of expression patterns involved, the biological programs modulated in each of them, and the potential core elements involved in the regulation of such time-dependent patterns. In this work, we have utilized this type of multidimensional bioinformatics strategy to determine the age-dependent transcriptomal changes in CD34^+^ Itgα6^+^ SSCs. Our results indicate that the transcriptome of these cells is, in fact, highly dynamic along the age points interrogated. As a reference, our analyses have shown that approximately one third of the SSC transcriptome is highly dependent on that parameter. The dynamic gene subset of this transcriptome is also highly complex when considering the expression patterns exhibited by the differentially expressed genes, the number of genes involved in each of those dynamic subsets, and the type of biological functions linked to each of them. In line with this, our soft-clustering analyses have revealed that the age-dependent transcriptome of these cells can be subdivided into 14 different subsets according to their specific pattern of expression during the time periods analyzed. Further adding to this complexity, we found subsets that are age period-specific (e.g., subsets #1, #1.2, #2, #6, and #−6) (Figure 2) whereas others display two or more differential expression peaks during the entire time period interrogated (e.g., the bimodal subsets #1.5, #2.3.5, #3.5, #5.3, #−4.2, #−4.6) (Figure 2). These latter gene subsets are also quite variable depending on the relative number of genes involved in each expression peak (e.g., compare subsets #2.3.5 and #3.5 in Figure 2). This high level of variegation is probably due to the presence of multiple genes and biological functions that are identified using this unbiased approach, given that the level of complexity decreases significantly when analyses are performed using gene signature collections selected according to well-defined biological and signaling criteria (Figure 3D).

From a biological point of view, our analyses indicate that each of those differentially expressed gene subsets can be correlated with specific biological programs. In this context, we found that the “very early” and the “late” time-points selected in our experimental approach are the most homogenous from a functional point of view. Thus, the “very early” time-point gene signature share many molecular features that correlate with increased cell proliferation rates. By contrast, the “late” time-point exhibits molecular fingerprints associated with reduced stem cellness, increased cell differentiation, and aging. These data are interesting because they suggest that the transcriptomal features typical of “aging” SSCs arise much earlier than previously anticipated and, in fact, even earlier than the functional deterioration that is only observed in SSCs six months later in mice [6]. The “early” time-point also shows functional features consistent with the cessation of the proliferative status previously seen in the “very early” stage. However, the gene subsets observed in the “middle” time-points the are more heterogeneous as they include a variety of disparate metabolic, cell differentiation, and signaling pathways. This age interval is also characterized by reduced numbers of dynamic genes when compared to the rest of time-points included in our analyses.

It is worth noting that the age-dependent biological programs found in the foregoing analyses are also detected when the microarray data are processed using independent, pathway-centered coexpression analyses. Furthermore, these latter in silico studies have unveiled additional functional features of those programs. For example, we saw that the proliferative condition found at the “very early” time-point also correlates with elevated levels of Hedgehog- and E2F-linked gene signatures as well as with reductions in the expression levels of genes associated with TGFβ and Notch1 signaling (Figure 3C,D). By contrast, the differentiated and aging features of the SSCs detected in the “late” time-point go in parallel with the elevation of cell adhesion-, apoptosis-, TNFα-, Wnt-, Notch1-, proliferation-, and inflammation-related gene signatures (Figure 3C,D). Importantly, our experiments were designed to interrogate the “coding” transcriptome using the Affymetrix microarray platform. It is likely that the application of RNA sequencing techniques could provide further functional insights on the age-dependent changes in the transcriptome and functions of SSCs such as, for example, alterations in noncoding RNAs or in patterns of splicing of specific mRNAs.

It is likely that the transcriptomal programs characterized using genome-wide expression analyses contain multiple bystanders that play no relevant processes in SSC biology. Separating the wheat from the chaff in these transcriptomal programs would be difficult unless functional screenings are conducted. However, one avenue to tackle this issue in silico is to carry out weighted correlation network analyses to identify transcriptional hubs in the expression networks characterized. Using this approach, we reduced the initial collection of 11,123 differentially expressed genes to a much smaller subset of 1087 genes that can be considered as hubs in this age-dynamic transcriptomal process (9.8% of the total dynamic transcriptome). This collection could be further decreased if needed just by increasing the statistical and a priori identification criteria used in these correlation network analyses. Several lines of evidence suggest that this method provides a good picture of the potentially relevant genes involved in each of the dynamic gene subsets. Those include (i) the observation that the gene hub collection recapitulates well the age-dependent fluctuations of the biological processes found upon the functional annotation of the whole dynamic SSC transcriptome. (ii) That a significant fraction (34.8%) of the identified gene hubs has been previously linked to SSC-related processes [6,9,10]. It would be interesting to characterize in the near future the functional relevance of the remaining 65.2% gene hubs that, up to date, have not been characterized as yet in the context of SSC biology.

Another issue bound to this type of study is the identification of the master regulators of the transcriptomal changes observed. Prima facie, it could be argued that the upstream transcriptional factors in charge of such process had to behave as transcriptional hubs themselves (unless their regulation takes place at the posttranscriptional level). Consistent with this view, we detected a significant number of transcriptional factors in our age-regulated gene hubs (Figure 5A). Unfortunately, most of those proteins are poorly characterized as yet and, therefore, are not well represented in the datasets commonly used to identify enriched DNA binding sites in the promoter regions of differentially expressed genes. As a result, we could not validate those data using this type of independent analyses. A way to circumvent this problem is just focusing on the in silico identification of transcriptional factors whose DNA binding motifs are enriched in the promoter regions of the age-regulated gene subsets. Using this approach, we did find a number of transcriptional factors that can be potentially involved in the age-dependent regulation of the SSC transcriptome (Figure 5B). The detection of some of these transcriptional factors is consistent with some of the age-dependent programs and SSC biology described in this work and previous studies, respectively. However, we have also identified some transcription factors that had not been connected to SSC functions as yet. It would be important to carry out wet-lab analyses to establish whether such factors do contribute to either the homeostasis or the aging of SSCs. Interestingly, we could not find consistent age-dependent variations in the mRNA levels of most of these transcriptional factors (data not shown). This suggests that their stimulation takes place at either the translational or posttranslational levels.

Given that the main focus of this study was the characterization of the age-dependent evolution of the SSC transcriptome under normal homeostatic conditions, we have not characterized the transcriptome of those cells in aging mice. Up to now, this issue has been addressed by a study that utilized SSCs from fully aged (18-month-old) mice [6]. As indicated above, our study indicates, however, that the molecular features of those aging SSCs are already seen in earlier age periods. An issue that remains to be studied in the stem cell field is whether such “aging” features become further aggravated in much older mice (due to changes in gene expression levels or to engagement of new, aging-associated gene expression programs). This issue can be readily analyzed using the in silico tools described in the present work once the samples for such age periods become available. The same applies for the monitoring of gene expression changes that take place during the development of skin diseases such as, for example, cancer or psoriasis.

It is important to bear in mind that the age-dependent transcriptomal changes observed in this study do not necessarily have to be mediated by some internal clock of the SSCs. Indeed, some of these changes can be elicited by either paracrine or cell-to-cell signals from the niche. As a token, the upregulation of gene signatures associated with the activation of the Jak-Stat signaling pathway detected in aging SSCs is known to be engaged by cytokines released from the surrounding tissue [6]. It is likely therefore that the age-dependent fluctuations in gene expression seen in this work could be result of the combination of both intrinsic and extrinsic signals that regulate SSC biology.

In addition to providing a large angular view of the age-dependent evolution of the SSC transcriptome, this work has described a computational pipeline that can be easily implemented to characterize, integrate, and extract functional information from the dynamic changes in transcriptomes linked to multiple biological time-points and/or experimental conditions.

## Supplementary Materials

The following are available online at https://www.mdpi.com/2073-4409/9/1/165/s1. Supplementary Figure S1. Time-driven expression patterns in mouse SSCs. Combined gene ontology analyses of very early, early, middle, and late expression patterns shown in Figure 2. Bar orientation indicates positive (up) and negative (down) enrichment against baseline. Table S1. Age-regulated subsets of the transcriptome of SSCs. The peak position (1 = 18-day-old, 2 = 30-day-old, 3 = 2.5-month-old, 4 = 4-month-old, 5 = 6-month-old, 6 = 12-month-old), probeset, and gene symbol are indicated. Table S2. Intramodular connectivity of the epidermal stem cell dynamic expression patterns. The peak position (1 = 18-day-old, 2 = 30-day-old, 3 = 2.5-month-old, 4 = 4-month-old, 5 = 6-month-old, 6 = 12-month-old), the gene symbol, and the intramodular connectivity score are indicated.
