# Supplementary material for: In Silico Analysis of the Age-Dependent Evolution of the Transcriptome of Mouse Skin Stem Cells
Source: Cells. 2020 Jan 9;9(1):165. doi: 10.3390/cells9010165 (PMC7016981; doi:10.3390/cells9010165)
Supplement: Supplementary file 1 [file cells-09-00165-s001.zip › Bustelo.SuppInfo.Cells.V2.pdf]

Supplemental Information for

In silico analysis of the age–dependent evolution of the transcriptome of mouse skin stem cells

by

L. Francisco Lorenzo–Martín and Xosé R. Bustelo*

*To whom correspondence should be addressed. E–mail: xbustelo@usal.es

This PDF file includes:
(1) Supplemental Figure S1 and legend (pages 2)

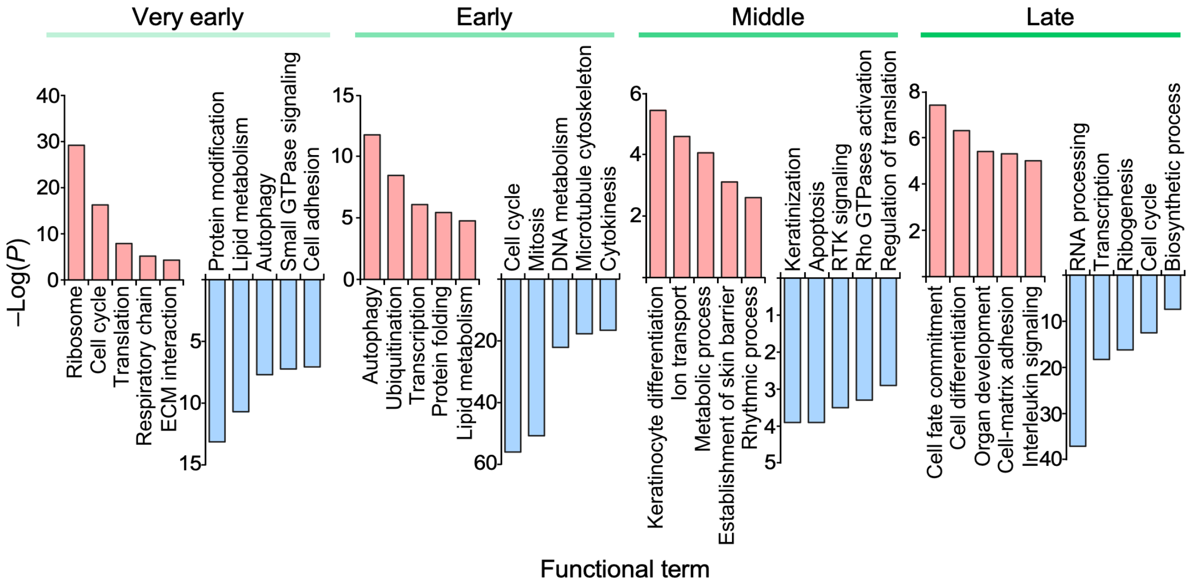

Figure S1. Time–driven expression patterns in mouse SSCs. Gene ontology analyses of the indicated gene expression patterns (top) derived from the analyses shown in **Figure 2**. Bar orientation indicates positive (up) and negative (down) enrichment against baseline.
